# Supplementary material for: Impact of household food insecurity and nutrition on depression and anxiety symptoms among adolescents living in rural Pakistan
Source: Glob Ment Health (Camb). 2025 Jul 10;12:e79. doi: 10.1017/gmh.2025.10006 (PMC12322787; doi:10.1017/gmh.2025.10006)
Supplement: Campisi et al. supplementary material 1 — Campisi et al. supplementary material [file S205442512510006Xsup001.docx]

**Impact of food insecurity and nutrition on depression and anxiety symptoms among adolescents living in rural Pakistan.**

*Susan C Campisi ^a,b,c,d^, [susan.campisi@sickkids.ca](mailto:susan.campisi@sickkids.ca)

*Florence Perquier ^a^, florence.perquier@camh.ca

Yaqub Wasan ^f^, yaqub.wasan@aku.edu

Sajid B Soofi ^f^, [sajid.soofi@aku.edu](mailto:sajid.soofi@aku.edu)

Daphne Korczak ^b, g^, daphne.korczak@sickkids.ca

Suneeta Monga ^b, g^, suneeta.monga@sickkids.ca

Peter Szatmari ^a, b, g^, peter.szatmari@utoronto.ca

Zulfiqar A Bhutta ^d, f^, zulfiqar.bhutta@aku.edu

*Indicates equal contribution

**Author Affiliations**

^a^ Cundill Centre for Child and Youth Depression, Centre for Addiction and Mental Health, 80 Workman Way, Toronto, Ontario, Canada

^b^ Department of Psychiatry, Hospital for Sick Children, 525 University Avenue, Toronto, Ontario, Canada

^c^ Nutrition and Dietetics Program, Clinical Public Health Division, Dalla Lana School of Public Health, University of Toronto

^d^ Centre for Global Child Health, Hospital for Sick Children, Peter Gilgan Centre for Research and Learning, 686 Bay Street, Toronto, Ontario, Canada

^e^ Department of Behavioural Neurosciences & Psychiatry, McMaster University, 1400 Main St W, Hamilton, Ontario, Canada

^f^ Centre of Excellence in Women and Child Health, Aga Khan University Stadium Road, PO Box 3500, Karachi, Pakistan

^g^ Department of Psychiatry, Temerty Faculty of Medicine, University of Toronto, Toronto, Ontario, Canada

**Corresponding Author:** Susan Campisi susan.campisi@sickkids.ca

**Supplementary Table 1**. Weekly Food Groupings Mean Intake, by sex

| Food Grouping | Household Intake/Week  (mean [SD]) | | *p-*value |
| --- | --- | --- | --- |
|  | Male | Female |  |
| Foods made from grains | 6.99 [0.23] | 6.99 [0.20] | .609 |
| White roots, tubers and plantains | 4.69 [1.52] | 4.61 [1.42] | .065 |
| Pulses (beans, peas and lentils) | 1.94 [1.29] | 2.00 [1.32] | .522 |
| Nuts and seeds | 0.23 [1.04] | 0.20 [0.95] | .842 |
| Milk and milk products | 6.89 [0.81] | 6.90 [0.79] | .731 |
| Organ meat | 0.21 [0.77] | 0.24 [0.78] | .274 |
| Meat, poultry | 1.02 [1.14] | 1.12 [1.28] | .533 |
| Fish and other seafood | 0.52 [1.03] | 0.52 [0.86] | .303 |
| Eggs | 1.62 [2.21] | 1.54 [2.07] | .966 |
| Dark green leafy vegetables | 3.25 [1.87] | 3.38 [1.75] | .169 |
| Vitamin A-rich vegetables | 4.50 [1.62] | 4.36 [1.57] | **.031** |
| Vitamin A-rich fruits | 1.93 [2.09] | 1.89 [2.15] | .456 |
| Other vegetables | 4.34 [1.54] | 4.25 [1.55] | .160 |
| Other fruits | 1.78 [2.05] | 1.74 [2.10] | .472 |
| Oil and fats | 6.92 [0.73] | 6.96 [0.49] | .159 |
| Sweets and sugar | 6.95 [0.66] | 6.97 [0.46] | .135 |
| Condiments | 6.62 [1.50] | 6.74 [1.24] | .091 |
| Other beverages and foods | 6.97 [0.39] | 6.94 [0.57] | .157 |
